# Implementation and process analysis of pilot scale multi-phase anaerobic fermentation and digestion of faecal sludge in Ghana

**DOI:** 10.12688/gatesopenres.12754.1

**Published:** 2017-11-06

**Authors:** Justin Shih, Ato Fanyin-Martin, Edris Taher, Kartik Chandran

**Affiliations:** 1 Earth and Environmental Engineering, Columbia University, New York, NY, 10027, USA; 2 Chemical Engineering,, Kwame Nkrumah University of Science and Technology, Kumasi, Ghana

**Keywords:** faecal sludge, anaerobic fermentation and digestion, volatile fatty acid, chemical oxygen demand

## Abstract

**Background.  **In Ghana, faecal sludge (FS) from on-site sanitation facilities is often discharged untreated into the environment, leading to significant insults to environmental and human health. Anaerobic digestion offers an attractive pathway for FS treatment with the concomitant production of energy in the form of methane. Another innovative option includes separating digestion into acidogenesis (production of volatile fatty acids (VFA)) and methanogenesis (production of methane), which could ultimately facilitate the production of an array of biofuels and biochemicals from the VFA. This work describes the development, implementation and modeling based analysis of a novel multiphase anaerobic fermentation-digestion process aimed at FS treatment in Kumasi, Ghana.

**Methods.  **A pilot-scale anaerobic fermentation process was implemented at the Kumasi Metropolitan Assembly’s Oti Sanitary Landfill Site at Adanse Dompoase.  The process consisted of six 10 m ^3^ reactors in series, which were inoculated with bovine rumen and fed with fecal sludge obtained from public toilets.  The performance of the fermentation process was characterized in terms of both aqueous and gaseous variables representing the conversion of influent organic carbon to VFA as well as CH _4_.  Using the operating data, the first-ever process model for FS fermentation and digestion was developed and calibrated, based on the activated sludge model framework.

**Results and Conclusions.  **This work represents one of the first systematic efforts at integrated FS characterization and process modeling to enable anaerobic fermentation and digestion of FS. It is shown that owing to pre-fermentation of FS in public septage holding tanks, one could employ significantly smaller digesters (lower capital costs) or increased loading capabilities for FS conversion to biogas or VFA. Further, using the first-ever calibrated process model for FS fermentation and digestion presented herein, we expect improved and more mechanistically informed development and application of different process designs and configurations for global FS management practice.

## Introduction

In Ghana, faecal sludge (FS) from on-site sanitation facilities is often discharged untreated into drainage ditches, inland waters, and the Atlantic Ocean, leading to deteriorating water quality and increasing the spread of gastro-intestinal infections ( Cofie *et al*., 2006). While conventional wastewater treatment (primary, secondary, and tertiary) has proven to be effective in developed countries, the associated high energy demands with such treatment models may be cost prohibitive for developing countries to sustain ( Murray & Buckley, 2010). One promising strategy for sustained FS treatment is to consider any potential end uses of FS that can offer incentives through resource recovery ( Diener *et al*., 2014). Anaerobic digestion offers an attractive pathway for FS treatment with the concomitant production of energy in the form of methane. Another innovative option includes separating digestion into acidogenesis (production of volatile fatty acids (VFA)) and methanogenesis (production of methane), thereby facilitating the production of an array of biofuels and biochemicals from the VFA ( Chandran, 2014). 

In order to determine the feasibility of these treatment technologies, numerous FS characterization studies have been performed in the last two decades ( Bassan *et al*., 2013; Kengne *et al*., 2011; Koottatep *et al*., 2005), including those specifically evaluated in Ghana ( Cofie *et al*., 2006; Strauss *et al*., 1997). These studies provide a baseline of FS characteristics, often focusing on chemical oxygen demand (COD), biochemical oxygen demand, solids (total solids, total volatile solids, total suspended solids, or volatile suspended solids), COD fractionation ( Lopez-Vazquez *et al*., 2014), pH, or ammonia-nitrogen. However, there is a general lack of FS characterization beyond conventional parameters.

This absence of more detailed characterization presents a challenge for determining clear design guidelines for resource or energy recovery. For example, in Ghana, anaerobic digesters accepting FS are typically sized for a solids retention time (SRT) of 15–60 days for pathogen reduction ( Bensah & Brew-Hammond, 2010). In the United States, the design SRT of sewage sludge digesters for biosolids treatment is dependent on the desired biosolids Class ( USEPA, 2006), but is usually higher than 20 days for single stage mesophilic digesters ( Song *et al*., 2004) to meet pathogen and volatile solids reduction requirements. However, if methane production and hygienization are concurrent project goals, it may not be necessary for them to occur in the same process ( Astals *et al*., 2012). Rather, by pairing individual goals with specific processes as part of a multi-stage system, the improvements in efficiency may result in more efficient, lower cost systems.

Given that initial capital cost is a key limiting factor to biogas dissemination, especially in developing nations ( Bensah & Brew-Hammond, 2010), any reductions in anaerobic digester sizing (where applicable) may assist in more efficient FS management and improved systems for biogas production and usage. Smaller digesters also open up opportunities for installation in dense urban areas with space limitations. By determining the optimal SRT for recovery of specific end products, process designers and engineers can perform a more accurate cost benefit analysis for resource recovery in specific systems. Accordingly, this work describes the process development, implementation and modeling based process analysis of a novel multi-phase anaerobic fermentation-digestion process aimed at FS processing in Kumasi, Ghana.

## Methods

### Study background and facility layout

In partnership with the Kumasi Metropolitan Assembly, pilot-scale anaerobic fermentation and digestion of FS was conducted at the Kumasi Metropolitan Assembly’s Oti Sanitary Landfill Site at Adanse Dompoase (Dompoase), the disposal point for FS from Kumasi and bordering neighborhoods. FS from septic tanks, aqua privies, latrines, or unsewered public toilets ( Cofie *et al*., 2006; Kuffour *et al*., 2013) is regularly collected by vacuum trucks and discharged into a series of waste stabilization ponds. FS from public toilets was exclusively used in this study since it represents the largest toilet usage category (34.6% of Ghanaians) as reported by the 2010 Ghana Census ( Ghana Statistical Service, 2012). Public toilet FS includes both wet (utilizing water for flushing) and dry (not utilizing flush water) toilet blocks.

**Figure 1.**
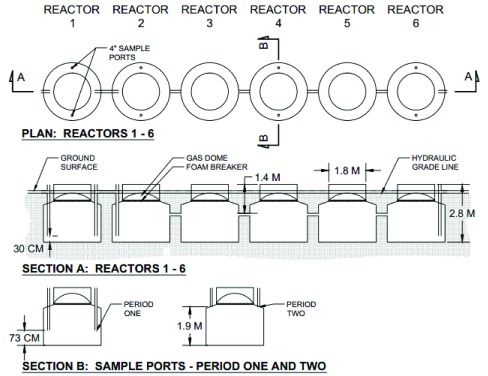
Layout of the faecal sludge anaerobic fermentation and digestion facility in Kumasi, Ghana.

The multi-phase anaerobic fermentation-digestion process consisted of six, 10 m ^3^ concrete anaerobic reactors in series (labeled R1- R6 in Figure 1). The anaerobic reactors were buried and operated unheated with an average liquid temperature of 28°C. Two sample ports each were installed on R1, R2, R4, and R6 for initial inoculation, mixing, and liquid sampling. FS was received from vacuum trucks in batches of 5–10 m ^3^ and fed in turn into a 1.2 m × 1.2 m receiving box (0.3 m depth) through a 1 cm metal screen, which typically retained clothing material, condoms, plastic bags, and plastic wrappers. The variable batch volume was due to the maximum capacity and the load volume carried by the specific trucks, which varied by delivery. After FS was received, it was pumped into a 10 m ^3^ holding tank (round, 2.4 meters tall) at grade before gravity fed into the first reactor in the series of six reactors ( Figure 1). The 10 m ^3^ holding tank allowed for a defined volume to be loaded into the system.

### Loading periods – fed batch loading

The monitoring data for this study was separated into two distinctive loading periods: Loading Period One (days 1–70), beginning on April 22, 2013, and loading Period Two (days 71–156). Period One had an average daily loading volume of 2,806±3,484 L, resulting in an average hydraulic retention time (HRT) of 3.6 days per reactor. Period Two had an average daily loading volume of 4,413±1,540 L, resulting in an average HRT of 2.3 days per reactor. In general, Period Two had higher overall loading volumes and less loading volume variability ( Figure S1), due to more consistent loading days throughout the week (6.4 days per week vs. 3.5 days per week, on average over the two respective loading periods). Period One was also characterized by higher precipitation than Period Two, which in combination with unpaved roads led to difficulties in receiving influent at the site, which resulted in increased loading volumes to compensate during Period One.

During Period One, the sample ports extended until 73 cm from the bottom of the reactor, and the 2” mixing pipes extended to the bottom of the reactor. However, due to increased clogging of gas sample lines, these sample ports were shortened to the elevation of the reactor shoulder for Period Two. This allowed the discharge pipe to direct FS towards the foam breakers, potentially preventing solids accumulation that was passing through the foam breakers and clogging the gas sample lines.

Before the start of both Period One and Period Two, FS was transferred between the two rows of reactors to allow for physical removal of any rumen, settled solids, and upper crust layer from the active liquid stream. This ensured that any organic contributions during the Loading Periods were due to incoming FS and not any previous solids accumulation. 

### Start-up and process operation and monitoring

The inoculation and start-up period spanned 144 days (pre-Day 1 to pre-Day 144), beginning on November 29 ^th^, 2012. Bovine rumen was used to inoculate the reactors. Rumen is a well-documented inoculum for anaerobic digestion ( Hu & Yu, 2005; Lopes *et al.*, 2004) and was sufficiently available from the Kumasi Abattoir. On pre-Day 1, R1 was loaded with 8,500 L of FS and inoculated with 1,500 L of bovine rumen (separated from blood and carcass). All reactors were filled with FS on pre-Day 51, and additional rumen (1,500 L per reactor) was loaded through the sample ports into R1, R2, R4, and R6 on pre-Day 71. FS loading was ramped-up starting from 1,000 L/day (on pre-Day 91), increasing to 2,500 L/day (on pre-Day 106), and finally reaching 4,000 L/day (on pre-Day 131). 

The reactors were mixed five days a week during Period One and seven days a week during Period Two by pump circulation with a gasoline powered 2” centrifugal pump (standard trash pump) at 340 L/min for 16 minutes per day (clockwise for 8 minutes and counter-clockwise for 8 minutes). On sampling days, the first operation performed was gas flow measurement and gas analysis through the gas sample ports installed onto R1, R2, R4, and R6. Plastic flexible tubing was used to connect the sample port to an inverted graduated cylinder, allowing for gas flow measurement through the volume displacement method. Starting from Day 85, a GFM416 gas analyzer (Gas Data Ltd., Coventry, UK) was used to measure CH _4_, CO _2_, and H _2_S in the off gas.

Reactor samples were taken twice a week from R1, R2, R4, and R6 through the sample ports immediately after reactor mixing. A 237 mL Bacon Bomb sampler (Clarkson Laboratory and Supply Inc., Chula Vista, CA) was lowered through the sample port to take one sample 20 cm from the bottom of the reactor and one sample 30 cm above the first sample from both sample ports. These four samples were mixed together in a 1 liter plastic container and stored in a cooler until transport to the lab.

Laboratory analysis of reactor samples was performed at Kwame Nkrumah University of Science and Technology in Kumasi, Ghana. Total chemical oxygen demand (tCOD, HACH Method 8000) and total volatile fatty acids (VFA, HACH Method 8196) were tested twice a week, while total suspended solids (TSS, US EPA Method 160.2), volatile suspended solids (VSS, US EPA Method 160.4), alkalinity (US EPA Method 310.1), pH (US EPA Method 150.1), and ammonia-nitrogen (NH _3_-N, US EPA Method 350.3) were tested once a week. Related methods pertaining to analysis of wastewater-related samples can also be found readily at https://www.epa.gov/cwa-methods.

### Process modeling

The pilot facility was dynamically simulated using BioWin 4 (EnviroSim, Oakville, CA) through input of measured influent loads and calibration with measured reactor results. The Biowin setup consisted of six anaerobic digester modules in series each defined with a 10,000 liter volume, depth of 1.8 meters and head space volume of 1,500 L under 103 kPa to represent field reactors. Daily loading volumes, tCOD, pH, alkalinity, and inorganic suspended solids (ISS) were directly inputted from measured influent values. A total kjeldahl nitrogen (TKN)/COD ratio of 0.16 TKN/COD was determined by calculating a ratio of NH _3_-N/COD and scaling by 33% to balance nitrogen in the influent, and a total phosphorus (TP)/COD ratio of 0.04 was determined from measured phosphorus in Kumasi FS. Nitrate N and dissolved oxygen were left as 0 mgN/L and 0 mg/L, respectively, given the anaerobic conditions of toilet systems, and calcium and magnesium were left as default values of 80 mg/L and 15 mg/L. Finally, given that FS vacuum trucks often do not remove all settled material from toilet systems, “settled” wastewater COD fractionation values were used as initial values.

The calibration methodology focused on identifying and limiting model adjustments to a minimal number of significant, justifiable parameters. Throughout the calibration, central composite response surface designs were created using Minitab 17 (State College, PA) to evaluate the significance of variables, determine optimal variable inputs (through the response optimizer tool) and minimize the number of model runs required to evaluate multiple variables. Optimal variable inputs were evaluated by their composite desirability, which is calculated as the weighted geometric mean of individual desired responses (measured from 0 to 1, 1 as ideal). Given the number of variables and reactors, model development was based on average simulated and average observed values over both loading periods.

**Figure 2.**
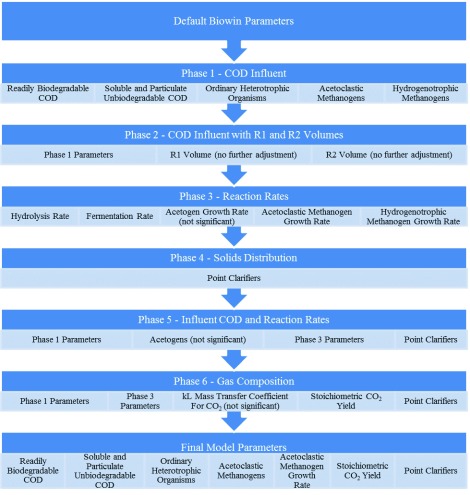
Model development chronology. COD, chemical oxygen demand.

The calibration process was conducted through a chronological series of hypotheses that primarily focused on VFA concentrations and gas production. VFA and gas production serve as benchmarks that characterize the stage or degree of anaerobic digestion, and they are also the target end products for this study. tCOD, % CH _4_, and % CO _2_ were also directly used for model calibration. All considered variables are documented through the following calibration phases ( Figure 2): 

Calibration Phase 1: COD influent parameters were the first to be adjusted due to COD fractionation reference values as well as expected microbial populations inherent in FS. A soluble unbiodegradable fraction (Fus) of 0.09 and particulate unbiodegradable fraction (Fup) of 0.47 was used ( Dangol *et al*., 2013) from a previous FS study. A full factorial central composite response surface design was created in Minitab for the following influent parameters and factor ranges: readily biodegradable (including acetate) g COD/ g total COD (Fbs, 0.05–0.2); ordinary heterotrophic organisms (OHO) COD fraction g COD/ g total COD (FZbh, 0.1–0.5); acetoclastic methanogen COD fraction g COD/ g total COD (FZbam, 0.0001–0.005); and hydrogenotrophic methanogen COD fraction g COD/ g total COD (FZbhm, 0.0001–0.005). 

Calibration Phase 2: Given that Biowin simulates the anaerobic digester modules as completely mixed reactors, increased reactor sizes for R1 and R2 were explored in addition to influent parameters to simulate longer solids retention time in the upstream reactors. A half factorial central composite response surface design was created for the following influent parameters and factor ranges: Fbs (0.03–0.27), FZbh (0.01–0.1), FZbam (0.0001–0.01), FZbhm (0.0001–0.01), R1 volume (10,000–30,000 L), and R2 volume (10,000–30,000 L). 

Calibration Phase 3: Reaction rates were considered next, focusing on the key aspects of anaerobic digestion: hydrolysis, acidogenesis, acetogenesis, and methanogenesis. A half factorial central composite response surface design was created for the following reaction rates and factor ranges: hydrolysis rate (0.36–3.84), maximum specific growth rate of OHO (0.07–3.13), maximum specific growth rate of acetogens (0.07–0.43), maximum specific growth rate of acetoclastic methanogens (0.07–0.53), and maximum specific growth rate of hydrogenotrophic methanogens (0.07–2.73) on VFA and gas production. Note: Biowin utilizes a 0.2 anaerobic hydrolysis factor to account for reduced hydrolysis rates under anaerobic conditions. To further explore the extent of reaction rate adjustments, local rate parameters were grouped into R1–R2 and R3–R6 due to the hydraulic connection locations, as well as the solids distribution profile. 

Calibration Phase 4: Volume-less point clarifiers were added to recirculate solids within each reactor to simulate the observed solids concentrations therein. The point clarifiers allow solids to be concentrated or diluted depending on the flow split ratio and solids removal percentage. These point clarifier settings were iteratively adjusted to calibrate tCOD concentrations.

Calibration Phase 5: After determining which COD fractionation values, influent microbial groups, and reaction rates significantly affected VFA and gas production, these variables (along with point clarifiers) were simultaneously considered for calibration. Additionally, the simulated microbial population ratios in R4 and R6 were considered as reference values for the influent conditions to reflect a microbial distribution where methanogenesis is active and stable.

Calibration Phase 6: Several alternatives were investigated to calibrate the % CH _4_ and % CO _2_ content in the off gas, including: lowering the hydrogenotrophic methanogen growth rate (along with increasing the acetoclastic methanogenic maximum specific growth rate to compensate for lower gas production), increasing the hydrolysis rate (to promote subsequent CO _2_ production during fermentation), lowering the readily biodegradable COD content in the influent to reduce initial gas production spikes in upstream reactors, increasing the gas-liquid mass transfer coefficient for CO _2_, and finally increasing the stoichiometric CO _2_ yield (moles of CO _2_ produced per mole of acetate formed) for OHO.

For the final model configuration, each parameter per reactor was evaluated (using Microsoft Excel 2010) by determining the root mean square error, as well as the Nash-Sutcliffe coefficient to consider the observed data variance.

### Gas production efficiency

Gas production efficiency per reactor (E _gas_) was calculated with Eq. 1, where Q _r_ is the gas production rate (mL off gas/day), HRT _r_ is the HRT per reactor, and COD _r_ is the tCOD (g) loaded into the reactor.

$${\text{E}}_{\text{gas}}=\frac{Q_{r}\times \text{HR}{\text{T}}_{r}}{COD_{r}}\;(Eq.1)$$

### Statistical analysis

Process modeling was conducted using BioWin 4 (EnviroSim, Oakville, CA). Minitab 17 (State College, PA) was employed to evaluate the significance of variables, determine optimal variable inputs (through the response optimizer tool) and optimize the number of model runs required to evaluate multiple variables. Added statistical analysis was conducted using built-in subroutines in Microsoft Excel 2010.

## Results and discussion

### Faecal sludge characterization

The observed high variability of tCOD and TSS in influent loads ( Table 1), stable pH of 8.1±0.3 and 7.9±0.2 (Period One and Two, respectively), and average NH _3_-N of 2039±1248 and 963±1,253 ppm (Period One and Two, respectively) were all consistent with previous FS characterization studies ( Bassan *et al*., 2013; Cofie *et al*., 2006; Kengne *et al*., 2011). Despite the high variability of tCOD, average influent VFA remained relatively low at 1151±839 and 1646±1214 mgCOD/L, and average alkalinity was measured as 170±94 and 141±73 mmol/L (Period One and Two, respectively), which is higher than observed in sewage (80 mmol/L, Forster-Carneiro *et al*., 2010). All of these measured parameters suggest that FS is partially degraded with low acidogenesis and some buffering capacity, which is to be expected given that the emptying frequency of FS varies from “weeks to years” ( Niwagaba *et al*., 2014).

**Table 1.** Measured data for Period One and Two. COD, chemical oxygen demand; VFA, volatile fatty acids.

	Total COD (mg/L)		Total Suspended Solids (mg/L)		Volatile Suspended Solids (mg/L)		Total VFA (mgCOD/L)	
	Period One	Period Two	Period One	Period Two	Period One	Period Two	Period One	Period Two
	Avg±SD (n)	Avg±SD (n)	Avg±SD (n)	Avg±SD (n)	Avg±SD (n)	Avg±SD (n)	Avg±SD (n)	Avg±SD (n)
Influent	14549±10863 (16)	31354±22740 (16)	13580±14553 (16)	18044±19463 (14)	11058±11755 (16)	14400±15179 (14)	1151±839 (18)	1646±1214 (21)
R1	12749±6879 (16)	23094±23306 (18)	7648±2235 (9)	19723±13610 (13)	5903±1434 (9)	14813±10508 (13)	808±343 (18)	1049±551 (23)
R2	15282±8323 (16)	19432±19472 (16)	10626±3756 (9)	19165±11195 (13)	8099±2759 (9)	14853±8258 (13)	733±269 (18)	936±461 (23)
R3								
R4	10882±9837 (16)	15440±15661 (18)	6869±6112 (9)	12258±7605 (13)	5304±4468 (9)	9331±5587 (13)	542±267 (18)	642±206 (23)
R5								
R6	6761±6252 (16)	7684±2876 (16)	2340±1494 (9)	7200±7173 (13)	1972±1153 (9)	5592±5204 (13)	514±180 (18)	652±278 (23)
	Off Gas Flow Rate (m3/hr)		pH		Alkalinity (mmol/L)		NH _3_-N (ppm)	
	Period One	Period Two	Period One	Period Two	Period One	Period Two	Period One	Period Two
	Avg±SD (n)	Avg±SD (n)	Avg±SD (n)	Avg±SD (n)	Avg±SD (n)	Avg±SD (n)	Avg±SD (n)	Avg±SD (n)
Influent			8.09±0.33 (8)	7.93±0.16 (7)	170±94 (8)	141±73 (7)	2039±1248 (11)	963±1253 (5)
R1	0.083±0.070 (15)	0.209±0.170 (23)	8.28±0.15 (5)	7.91±0.27 (8)	136±38 (5)	133±85 (8)	1728±774 (8)	1078±931 (6)
R2	0.057±0.039 (18)	0.144±0.097 (22)	8.24±0.10 (5)	7.90±0.30 (8)	162±25 (5)	140±59 (8)	1681±579 (8)	1210±963 (6)
R3		0.061±0.051 (13)						
R4	0.023±0.017 (17)	0.046±0.050 (23)	8.21±0.11 (5)	8.00±0.26 (8)	112±32 (5)	121±36 (8)	1310±537 (8)	979±619 (6)
R5		0.025±0.011 (13)						
R6	0.006±0.005 (15)	0.015±0.014 (23)	8.21±0.15 (5)	7.99±0.22 (8)	98±28 (5)	115±34 (8)	1244±575 (8)	915±551 (6)

tCOD concentrations in the influent FS were statistically higher (p=0.012) in Period Two (31,354±22,740 mg/L) than in Period One (14,549±10,863 mg/L). Influent total solids (TS) in Period One averaged 16,255±13,691 (n=16), while in Period Two they averaged 25,390±27,695 (n=13). One potential explanation for the differences in tCOD is that increased precipitation during Period One may have caused stormwater runoff to enter toilet systems and dilute the FS. Another supposition is that water is more available during the rainy season, and that FS truck operators are more willing to use water to aid in FS extraction from toilet systems. Although a previous study in Ouagadougou, Burkina Faso, did not find significant differences in FS sampled during the dry and rainy seasons ( Bassan *et al*., 2013), tCOD concentrations in Ouagadougou for latrines and septic tanks (10725±9508 mg/L) were also on average lower than in Kumasi. Given that FS is highly variable even within a community or township, it is difficult to discern the exact explanation for the differences across countries. Any planned FS treatment facility would greatly benefit from local FS characterization.

### Inoculation and reactor performance

Inoculation and start-up of the pilot reactors occurred over a total of 131 days. Any rumen addition or increase to the FS loading rate was followed by weeks of observation to prevent over accumulation of VFA and subsequent inhibition of methanogenesis ( Wang *et al*., 2009). However, throughout this entire start-up period, VFA averaged 427±305, 318±154, 286±119, and 268±129 mgCOD/L in R1, R2, R4, and R6, respectively ( Figure S2). Gas production was highest in the first reactor, averaging 0.070±0.065, 0.043±0.037, 0.029±0.016, and 0.018±0.011 m ^3^/hr in R1, R2, R4, and R6, respectively ( Figure S3). Additionally, the highest gas production values were not observed until higher FS loading rates. The lack of VFA accumulation in all reactors and generally limited gas production in downstream reactors suggest that the inoculation and ramp-up loading could have been implemented in a shorter time period.

Similar VFA and gas production trends were also observed during full operation of the reactors. While it was expected that VFA concentrations would be maximal in the upstream anaerobic reactors owing to higher fermentation kinetics relative to methanogenesis kinetics ( Metcalf & Eddy, 2003), VFA concentrations were actually highest in the influent and steadily decreased across the reactors. Gas production followed a similar trend, with maximal values in the first reactor decreasing to the last reactor. % CH _4_ and % CO _2_ remained consistent across the reactors. % CH _4_ averaged 63.5±2.1%, 64.3±2.1%, 63.6±2.0%, 64.2±2.3%, 62.8±1.8% and 63.2±2.0% and % CO _2_ averaged 24.4±2.6%, 24.3±1.8%, 21.9±1.9%, 24.0±1.9%, 23.2±2.5% and 24.3±2.1% in R1–R6, respectively. H _2_S (ppm) varied in the reactors, averaging 2159±474, 2444±299, 2795±442, 2791±305, 3012±460 and 2766±354 in R1–R6, respectively.

Given that the reactors in Kumasi, like many anaerobic digesters in the developing world, were unmixed, a generally decreasing solids distribution across successive reactors was observed in terms of COD, TSS, and VSS concentrations. Elevated concentrations in R2 may have been caused by conveyance of solids through localized flow paths at the bottom of R1 ( Figure 1), while the mid-level connections between R2–R6 encouraged settling in the reactors. Unmixed systems will inevitably be affected by the hydraulic conditions of the system, which presents a challenge in developing general performance guidelines. Accumulated solids may also eventually decrease the overall working volume of the reactor and should be addressed to improve process efficiency. 

Despite the potential impacts to gas production at the pilot facility, solids accumulation was not likely the governing factor. While a baseline level of gas production may be expected from retained solids, gas production in upstream reactors was still observed to drop down to very low levels, suggesting that retained solids do not considerably contribute to gas production in a sustained way. Rather, the fluctuating, yet considerable gas production in upstream reactors was due to the influent FS loads. The measured performance of this pilot facility represents one of the first efforts to document the fermentation and digestion of actual FS loads delivered by vacuum truck, allowing for an accurate consideration of the effect of FS variability. The processing modeling analysis presented next offers further insights into the measured trends in VFA and gas production from the FS treatment process.

### Process model calibration

Specifically, the following influent substrate fraction (Fbs) and biomass fractions (FZbh, and FZbam) were found to statistically significantly impact (p<0.05) VFA and gas production in all reactors, while the biomass fraction, FZbhm, significantly impacted for R1–R2 VFA and R1 gas production. Through adjustment of these parameters, it was found that Biowin either under-simulated VFA in downstream reactors or under-simulated gas production in upstream reactors. When increased R1 and R2 reactor volumes were explored to simulate solids accumulation through increased retention times (thus increasing gas production), both R1 and R2 volumes were found to be significant (p<0.05) for VFA and gas production, with the exception of gas production in R4. While Minitab’s response optimizer tool yielded numerous combinations of inputs for successfully simulating VFA and gas production, the ability to control R1 and R2 volumes resulted in highly heterogeneous options that did not provide insight into the distinguishing characteristics of FS or FS fermentation and digestion. Reactors volumes were subsequently left at 10,000 L to explore additional calibration options.

When hydrolysis, acidogenesis, acetogenesis, and methanogenesis reaction rates were adjusted for the system, Biowin continued to either under-simulate VFA in downstream reactors or under-simulate gas production in upstream reactors. The hydrolysis rate, maximum specific growth rate of OHO, and the maximum specific growth rate of acetoclastic methanogens significantly affected VFA and gas production, while the maximum specific growth rate for acetogens and the maximum specific growth rate for hydrogenotrophic methanogens (for gas production in R4 and R6) were not significant (p>0.05). While separate rate adjustments for R1–R2 allowed for increased congruence of simulated and observed VFA and gas production, reactions rates were ultimately left as global values for the entire system due to the lack of sufficient justification for rate differences among reactors.

**Figure 3.**
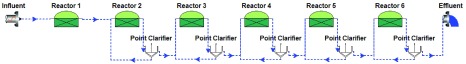
Final model configuration.

The solids distribution in the system was addressed by pairing the reactors with point clarifiers, which allowed for direct control of the solids concentration in each reactor. Given that Biowin’s anaerobic digester modules are complete-mix reactors, simulated tCOD concentrations were initially high in downstream reactors. Point clarifiers were added to R2, R4 and R6, and additional point clarifiers were eventually added to R3 and R5 in the final model configuration ( Figure 3). 

When the un-adjusted microbial population ratios were explored for R4 and R6, it was found that the concentration of OHO were roughly 2–3 times the amount of acetoclastic methanogens, which were in-turn roughly 3 times the amount of hydrogenotrophic methanogens. Hydrogenotrophic methanogens were found to be 10–20 times the amount of acetogens. Once the influent concentrations of these microbial groups along with COD fractionation values and reaction rates were considered together, congruence of VFA and gas production was obtained without adjusting local rate parameters. The best configuration from this phase retained default values for the hydrolysis rate (2.1 day ^-1^), fermentation rate (1.6 day ^-1^) and hydrogenotrophic methanogenesis rate (1.4 day ^-1^), and largely focused on adjusting FZbh, FZbam and the acetoclastic methanogenesis rate.

The final model consideration was the % CH _4_ and % CO _2_ in the off gas, which Biowin was initially over-simulating and under-simulating, respectively. While COD fractionation values and reactions rates were re-evaluated for gas composition, any adjustments negatively affected VFA and gas production congruence. Another strategy was to increase the gas-liquid mass transfer coefficient (kL) for CO _2_ to promote gaseous CO _2_, but this did not have detectable effect on % CO _2_ in the off gas. In order to better match the gas composition, the stoichiometric CO _2_ yield (moles of CO _2_ produced per mole of acetate formed) for OHO was ultimately increased. Due to acetoclastic methanogen dominance over hydrogenotrophic methanogens, a higher amount of acetate may be required in the system, thus increasing the stoichiometric yield above the Biowin default. It was found that an increase from the default 0.7 to 1.2 moles CO _2_/moles acetate successfully brought the simulated % CH _4_ and % CO _2_ in range with measured values.

### Final model configuration and performance

The calibrated model ultimately focused on the following influent parameters (all expressed as g COD/ g total COD): Fbs (0.09), Fus (0.09), Fup (0.47), FZbh (0.05), and FZbam (0.015). The acetoclastic methanogen maximum specific growth rate (0.1/day) and stoichiometric CO _2_ yield for OHO (1.2 moles CO2/moles acetate) were also adjusted from default values. Point clarifiers were implemented on R2–R6 with 25% solids removal and underflow ratios of 0.2, 0.35, 0.5, 0.625 and 0.75 for R2–R6, respectively.

Fbs was decreased from 0.27 to 0.09 g COD/ g total COD due to prior fermentation and digestion within the toilet systems. Accordingly, Fus and Fup were increased from 0.08 to 0.09 and 0.08 to 0.47 g COD/ g total COD, respectively, to represent higher concentrations of remaining unbiodegradable content. FZbh were increased from 0.01 to 0.05 OHO COD/total COD and FZbam (acetoclastic methanogens) from 0.0001 to 0.015 acetoclastic methanogen COD/total COD to reflect the high microbial concentrations inherent in FS, as demonstrated by maximal gas production in upstream reactors. While the influent concentrations of acetogens and hydrogenotrophic methanogen concentrations were adjusted in previous stages, due to their minor effects on VFA and gas production, they remained as default values. The acetoclastic methanogenesis maximum specific growth rate was decreased from 0.3 to 0.1 day ^-1^ due to the background methanogen population in the reactors, as well as potential ammonia inhibition of methanogenesis ( Yenigün & Demirel, 2013). 

**Figure 4.**
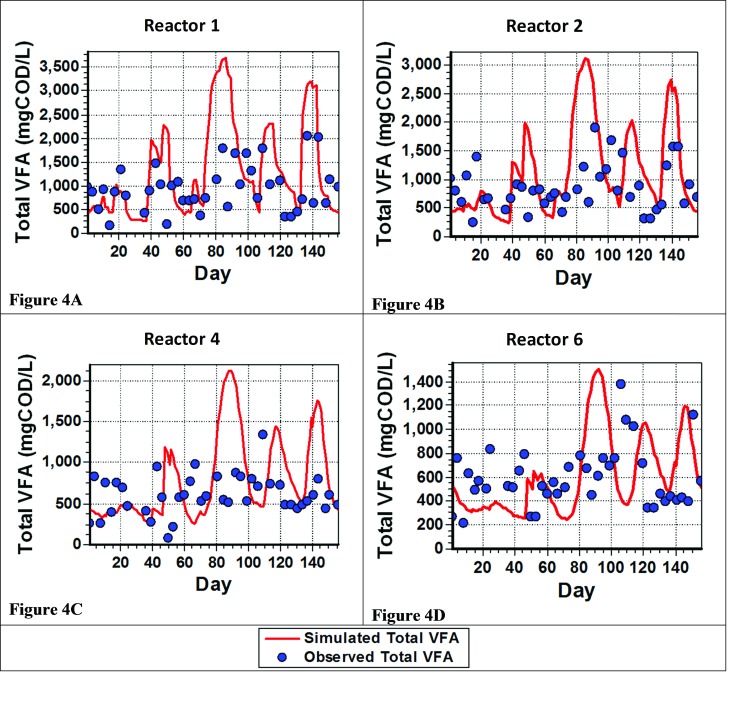
Total VFA, simulated and observed.

**Figure 5.**
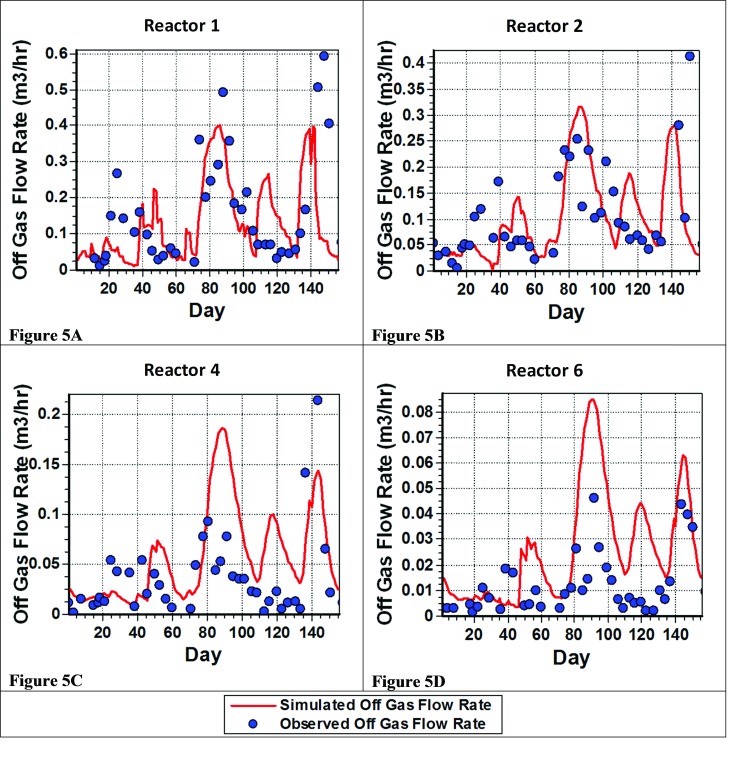
Off gas flow rate, simulated and observed.

Figure 4 and Figure 5 demonstrate congruence between simulated and observed output for VFA and gas production. The other parameters are documented in Figure S4– Figure S9. Table S1 documents the root mean square error and Nash-Sutcliffe coefficient for all parameters per reactor. While the Nash-Sutcliffe coefficients were close to 0 for TSS, VSS, tCOD (Reactors 1, 2, 4) and gas production (Reactors 1, 2, 4), the VFA coefficient ranged from -2 to -6. Given the variability in COD loading, Biowin simulated equivalent peaks in VFA, causing a higher residual variance than the observed data variance. While these values demonstrate that the model does not perfectly simulate all observed values, the model calibration process still resulted in overall congruity and allows for an evaluation of VFA and gas production potential.

Given the antecedent retention time in public toilet systems and subsequent methanogen population in FS, average influent VFA concentrations were less than 2,000 mg COD/L. Additionally, any further fermentation/digestion was shown to decrease VFA concentrations in the reactors. While these concentrations are lower in comparison to the potential of other feedstocks ( Chang *et al*., 2010), VFA can be harvested directly from FS without additional fermentation. Finally, given the variability of toilet systems, there is potential for producing higher concentrations of VFA from less stabilized sources, such as toilet systems that are emptied more frequently or toilet systems with lower water content and higher organic concentrations.

**Figure 6.**
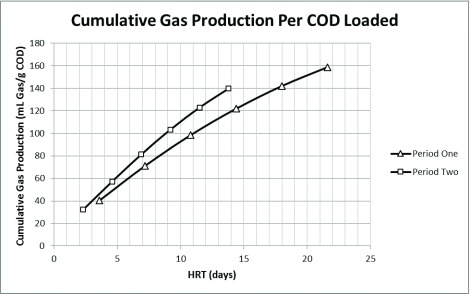
Cumulative gas production per COD loaded. COD, chemical oxygen demand; HRT, hydraulic retention time.

Gas production efficiency (mL off gas/g tCOD loaded) was calculated to be maximal in R1 and subsequently decreasing through R6 for both Period One and Two. In addition, gas production efficiency in Period Two (14.0±2.0, 10.9±1.2, 10.4±1.4, 9.6±1.5, 8.5±1.6, and 7.3±1.7 mL off gas/g tCOD loaded in R1–R6, respectively) was significantly higher in all reactors than efficiency in Period One (11.2±2.7, 8.5±1.6, 7.6±1.5, 6.5±1.5, 5.6±1.5, and 4.6±1.5 mL off gas/g tCOD loaded in R1–R6, respectively). Period Two, which had more consistent loading volumes, had roughly 15% more gas production after 14 days (140 vs 122 mL off gas/g tCOD loaded). The 75 ^th^ percentile of cumulative gas production for Period One and Two were obtained in 14 of 22 days and 9 of 14 days respectively ( Figure 6). These efficiencies were in range with a biomethane potential study in China, which found that FS produced 87 ml gas/g FS at 30°C ( Song *et al*., 2011). Period Two had a 1.23 tCOD/TS ratio, resulting in roughly 172 mL off gas/ g dry FS after 14 days. However, given the variability of FS beyond tCOD or TS, it is expected that biomethane potential studies would also yield variable results. When comparing with sewage sludge, one study in Sweden reported 271 m ^3^/ton VS loaded (271 mL/g VS), which at 76% VS/TS, is 206 mL/g dry sewage sludge ( Davidsson *et al*., 2008). While it is difficult to directly juxtapose these two waste streams considering the variability in each, the gas production efficiency of FS is comparable and has sufficient potential for further consideration.

This study presents one of the first calibrated FS fermentation and digestion process models. By exploring characterization of FS beyond tCOD and investigating reactor performance beyond conventional process indicators (pH, alkalinity, gas production, etc.), a more detailed and complete understanding is formed. FS, though often guided by wastewater or sewage sludge literature, is a very different feedstock. FS composition is highly variable, contains limited readily biodegradable COD due to prior degradation, and also harbors considerable OHO and methanogen concentrations. These singular characteristics have far-reaching implications for FS treatment design, which currently (erroneously) still relies on typical guidelines such as 20 day SRT for anaerobic processes at 30°C ( Metcalf & Eddy, 2003). Non-idealities such as settling also need to be considered for FS treatment process optimization, especially for highly concentrated FS. In order to effectively plan and evaluate these systems, all of these factors must be examined on a case by case basis.

## Conclusions

This work documents the start-up and operation of a pilot scale FS anaerobic fermentation and digestion system that processed FS from vacuum trucks in Kumasi, Ghana over a five month period. By utilizing measured field data from the pilot plant, this work details one of the first systematic efforts at integrated FS characterization and process modeling to enable anaerobic fermentation and digestion of FS. In addition to recording conventional process parameters for FS characterization, COD fractionation and existing microbial concentrations were also explored. In sum, it is shown that owing to pre-fermentation and digestion of FS in toilet systems, maximal VFA and gas production are expected at SRT values less than five days. While treatment modules such as biogas settlers are effective at maximizing gas production per COD load due to extended SRT (months or years) until desludging, these modules eventually become accumulated with digested sludge with lower gas production efficiency. There is potential for smaller digester units with lower SRT (for gas production) to be paired with a subsequent treatment step for potentially more cost effective hygienization, such as drying beds or infiltration based methods. This calibrated FS process model, along with the documented calibration approach, allows for future research into the expansion of biogas production from varying FS streams, as well as additional recovery of even higher-value resource outputs. 

## Data availability

Monitoring data during start-up and full operation are available on OSF: http://doi.org/10.17605/OSF.IO/WGRDX ( Chandran, 2017). Data are available under the terms of the Creative Commons Zero "No rights reserved" data waiver (CC0 1.0 Public domain dedication).

## Supplementary material

Supplementary File 1: File containing Table S1 (Statistical model evaluation); Figure S1 (Influent loading volumes); Figure S2 (Total volatile fatty acids, inoculation and ramp-up); Figure S3 (Off-gas flow rate, inoculation and ramp-up); Figure S4 (Total chemical oxygen demand, simulated and observed); Figure S5 (Total suspended solids, simulated and observed); Figure S6 (Volatile suspended solids, simulated and observed); Figure S7 (% CH _4_ and % CO _2_, simulated and observed); Figure S8 (pH, simulated and observed); Figure S9 (Alkalinity, simulated and observed).
