# Supplementary material for: Implementation and process analysis of pilot scale multi-phase anaerobic fermentation and digestion of faecal sludge in Ghana
Source: Gates Open Res. 2017 Nov 6;1:10. [Version 1] doi: 10.12688/gatesopenres.12754.1 (PMC5842369; doi:10.12688/gatesopenres.12754.1)
Supplement: Supplementary file 1 [file gatesopenres-1-13814-s0000.tgz › 5e1db487-ee6e-45d0-92f9-4b4cf51bd2bc.docx]

*Supplementary material*

**Implementation and Process Analysis of Pilot Scale Multi-phase Anaerobic Fermentation and Digestion of Faecal Sludge in Ghana**

**Justin Shih^1^, Ato Fanyin-Martin^2­^, Edris Taher^1^, Kartik Chandran^1,^***

^1^Earth and Environmental Engineering, Columbia University, New York, NY, 10027; ^2^Chemical Engineering, Kwame Nkrumah University of Science and Technology, Kumasi, Ghana; *Corresponding author: Email: [kc2288@columbia.edu](mailto:kc2288@columbia.edu)

Table S1: Statistical model evaluation. VFA, volatile fatty acids; COD, chemical oxygen demand.

|  | **Reactor** | **Root Mean Square Error** | **Nash-Sutcliffe** |
| --- | --- | --- | --- |
| Total VFA (mgCOD/L) | 1 | 978 | -3.13 |
|  | 2 | 784 | -2.91 |
|  | 4 | 600 | -5.77 |
|  | 6 | 437 | -2.27 |
| Off Gas Flow Rate (m3/hr) | 1 | 0.158 | -0.11 |
|  | 2 | 0.087 | 0.01 |
|  | 4 | 0.051 | -0.59 |
|  | 6 | 0.024 | -3.00 |
| Total COD (mg/L) | 1 | 15982 | 0.22 |
|  | 2 | 18583 | -0.57 |
|  | 4 | 14481 | -0.21 |
|  | 6 | 7078 | -1.23 |
| pH | 1 | 0.60 | -4.48 |
|  | 2 | 0.75 | -6.17 |
|  | 4 | 0.90 | -14.46 |
|  | 6 | 0.94 | -17.10 |
| Total Suspended Solids (mg/L) | 1 | 10155 | 0.28 |
|  | 2 | 8834 | 0.15 |
|  | 4 | 6733 | 0.11 |
|  | 6 | 4960 | 0.30 |
| Volatile Suspended Solids (mg/L) | 1 | 8411 | 0.15 |
|  | 2 | 6392 | 0.20 |
|  | 4 | 4923 | 0.12 |
|  | 6 | 3273 | 0.43 |
| Alkalinity (mmol/L) | 1 | 102 | -1.32 |
|  | 2 | 76 | -1.58 |
|  | 4 | 69 | -4.36 |
|  | 6 | 68 | -4.89 |
| NH_3_-N (ppm) | 1 | 2614 | -8.09 |
|  | 2 | 2419 | -8.83 |
|  | 4 | 2668 | -21.19 |
|  | 6 | 3056 | -29.44 |

Figure S1: Influent loading volumes.

Figure S2: Total volatile fatty acids (VFA) (mgchemical oxygen demand/L), inoculation and ramp-up.

Figure S3: Off gas flow rate (m3/hr), inoculation and ramp-up.

|  |  |  |  |
| --- | --- | --- | --- |

Figure S4: Total chemical oxygen demand (COD), simulated and observed.

|  |  |  |  |
| --- | --- | --- | --- |

Figure S5: Total suspended solids (TSS), simulated and observed.

|  |  |  |  |
| --- | --- | --- | --- |

Figure S6: Volatile suspended solids (VSS), simulated and observed.

|  |  |  |  |
| --- | --- | --- | --- |

Figure S7: % CH4 and % CO2, simulated and observed.

|  |  |  |  |
| --- | --- | --- | --- |

Figure S8: pH, simulated and observed.

|  |  |  |  |
| --- | --- | --- | --- |

**Figure S9: Alkalinity, simulated and observed.**
