# Supplementary material for: Implementation and process analysis of pilot scale multi-phase anaerobic fermentation and digestion of faecal sludge in Ghana
Source: Gates Open Res. 2017 Nov 6;1:10. [Version 1] doi: 10.12688/gatesopenres.12754.1 (PMC5842369; doi:10.12688/gatesopenres.12754.1)
Supplement: Peer review history

# Peer review: Referee response for version 1

**DOI:** 10.21956/gatesopenres.13814.r26048

**Published:** 2017-12-29

**Authors:** Zifu Li

**Affiliations:** 1 School of Energy and Environmental Engineering, Beijing Key Laboratory of Resource-Oriented Treatment of Industrial Pollutants, University of Science and Technology Beijing, Beijing, China

This work investigated the start-up and operation processes of a pilot scale faecal sludge anaerobic fermentation and digestion system. Beyond conventional parameters, additional parameters of faecal sludge characterization such as COD fractionation and existing microbial concentrations were also explored. Generally, it is very interesting and the results would be useful for further research and practical applications. However, it is suggested that some revisions should be done for better understanding and quality.

1.  A list of non-standard abbreviations should be added;
2. Maybe the schematic diagram and flowchart of entire system is needed for better description and understanding of the research objectives.
3. Page 5: All the parameters were measured instead of observed. The “observed” could be changed into “measured”.
4. Page 7: The “precipitation” is an easily confused word in this manuscript, which could be replaced by raining.
5. Page 7: “…localized flow paths at the bottom of R1…”, there is no bottom connection between R1 and R2 in Fig.1. It needs to be explained.  
6. Page 10: If the ordinate scales are kept same, the differences are more significant. 
7. It is also unusual that TSS in R1and R2 kept almost unchanged. The explanation should be given.
8. In the Methods, it is not clear how COD fractionations, and microbial population and concentrations were analysed. It is recommended that the analysis methods should be added.
9. A series of individual small digesters represents rather a plug flow model than a CSTR! After studying the published “supplementary material” it could be resumed that the technical-economical hydraulic retention time is about 40 days?!
10. Some typos should be corrected, such as “insults” should be “results”, “improved” should be “improve”, etc.

I have read this submission. I believe that I have an appropriate level of expertise to confirm that it is of an acceptable scientific standard.

# Peer review: Referee response for version 1

**DOI:** 10.21956/gatesopenres.13814.r26046

**Published:** 2017-12-06

**Authors:** Thammarat Koottatep, Tatchai Pussayanavin

**Affiliations:** 1 Environmental Engineering and Management, School of Environment, Resources and Development, Asian Institute of Technology, Bangkok, Thailand; 2 Asian Institute of Technology, Bangkok, Thailand

The paper presents the results of implemented of a pilot-scale anaerobic fermentation process in Ghana, the comparison of these data against full-scale units and finally the description and results of a model for the anaerobic process. The flow of the text and sections in the manuscript are sufficient. The title of the paper looks exciting; however, there is lack of some information in crucial aspects of the modeling exercise and validating results. I would then recommend this manuscript to go through a minor revision considering the following major aspects: 

1. In the process modeling, you have considered a lot of variables trying to modelling such a complex biological process as the anaerobic fermentation process. Biowin is a useful tool, however, the simulated results are different to the observed results. The VFA and off gas are overestimated according to figures 4-5. You have to refine your approach, because I can't see that you were able to simulate VFA/off gas production satisfactorily (Figure 4 and 5). Also, I would propose to have more data and validation phase to confirm the modelling.
2. Figure 2 is not so clear to understand the processes that you are modelling in each calibration phase. Is there a possibility to include a matrix notation to describe the processes (Petersen Matrix)?
3. Table 1, normally, the trend of average values of TCOD (mg/L) and off gas flow rate (m3/hr) was found to slightly decrease from R1-R6. However, the average TCOD value from R2 was higher than R1 and average off gas from R2 was lower than R1. Why conflict would occur among the results of performance R1 and R2? Please explain. 
4. Could you please explain with the evidence to prove the sentence included in page 9, which states that "Due to acetoclastic methanogen dominance over hydrogenotrophic methanogens, a higher amount of acetate may be required in the system, thus increasing the stoichiometric yield above the Biowin default”
5. Does the author take consider define/classifying the input COD fractionations/microbial populations of each R1-R2 instead of using only one reference value as the input?

We have read this submission. We believe that we have an appropriate level of expertise to confirm that it is of an acceptable scientific standard.
